# Chemical Approach to the Optimization of Conditions Using HS-SPME/GC–MS for Characterization of Volatile Compounds in *Eugenia brasiliensis* Fruit

**DOI:** 10.3390/molecules27154955

**Published:** 2022-08-04

**Authors:** Ana Luiza Coeli Cruz Ramos, Laiza Andrade Nogueira, Mauro Ramalho Silva, Ana Carolina do Carmo Mazzinghy, Ana Paula Xavier Mariano, Tássia Nunes de Albuquerque Rodrigues, Ana Cardoso Clemente Filha Ferreira de Paula, Angelita Cristine de Melo, Rodinei Augusti, Raquel Linhares Bello de Araújo, Inayara Cristina Alves Lacerda, Júlio Onésio Ferreira Melo

**Affiliations:** 1 Departamento de Alimentos, Faculdade de Farmácia, Campus Belo Horizonte, Universidade Federal de Minas Gerais, Belo Horizonte 31270-901, Brazil; 2 Departamento de Ciências Exatas e Biológicas, Campus Sete Lagoas, Universidade Federal de São João Del-Rei, Sete Lagoas 36307-352, Brazil; 3 Departamento de Nutrição, Pontifícia Universidade Católica de Minas Gerais, Belo Horizonte 30640-070, Brazil; 4 Departamento de Ciências Agrárias, Instituto Federal de Educação, Ciência e Tecnologia de Minas Gerais, Campus Bambuí, Bambui 38900-000, Brazil; 5 Departamento de Química, Campus Belo Horizonte, Universidade Federal de Minas Gerais, Belo Horizonte 35702-031, Brazil

**Keywords:** grumixama, SPME fibers, volatile organic compounds, sesquiterpene

## Abstract

Grumixama (*Eugenia brasiliensis* Lam.) is a native fruit of the Brazilian Atlantic Forest, belonging to the Myrtaceae family, which designatesthe most significant number of species with food potential. It stands out due to its phytochemical characteristics because of the presence of polyphenols and volatile organic compounds. Volatile compounds are substances released by foods that give off an aroma and influence flavor. Solid-phase microextraction is a technique that allows for low-cost, fast, and solvent-free extraction, has an affinity for numerous analytes, and is easily coupled to gas chromatography. The objectives of this work were to evaluate the efficiency of different fibers of SPME (solid-phase microextraction) in the extraction of volatile organic compounds from grumixama pulp; optimize a method for extraction time, temperature, and sample weight; and to determine the characteristic volatile profile of this fruit. For the extraction of volatile compounds, three fibers of different polarities were used: polar polyacrylate (PA) fibers, divinylbenzene/carboxyne/polydimethylsiloxane (DVB/CAR/PDMS) semipolar fibers, and polydimethylsiloxane/divinylbenzene (PDMS/DVB). Fourteen volatile organic compounds (VOCs) were identified by DVB/CAR/PDMS, six by PA, and seven by PDMS/DVB through solid-phase microextraction in the headspace mode (SPME-HS). Considering the total number of compounds identified, regardless of the fiber used, and the optimization of the method, *Eugenia brasiliensis* presented sesquiterpene fractions (85.7%, 83.3%, and 85.7% of total VOCs) higher than the monoterpene fractions (14.3%, 16.7%, and 14.3%) for DVB/CAR/PDMS, PA, and PDMS/DVB, respectively in its composition. In addition, it was possible to verify that the fiber DVB/CAR/PDMS presented a better efficiency due to the larger chromatographic area observed when the grumixama pulp was subjected to conditions of 75 °C, 2.0 g, and an adsorption time of 20 min.

## 1. Introduction

Brazil stands out for the immense biological diversity of its flora and is seen as one of the leading centers for the genetic diversity of fruit species worldwide. Although a wide variety of native fruit species is found in the Amazon and the Cerrado, the southern and southeastern regions also present a great richness in wild fruits. The Myrtaceae family stands out for designating the most significant number of species with food potential, which could be marketed in nature for use in the manufacturing of ice cream, juices, yogurts, liqueurs, desserts, cereal bars, jams, and jellies [1,2,3].

The grumixameira is a native tree of the Brazilian Atlantic Forest. The fruit of the grumixameira, *Eugenia brasiliensis* Lam., is approximately 2.0 cm in diameter. The skin is smooth, shiny, and has different colors [4,5]. In Brazil, grumixama is commercially used for the production of jellies, pies, and liqueurs in the southern and southeastern regions [6], harvested between November and February, and can also be used in public gardens and ecological restoration programs [5,7].

The chemical and physical composition and polyphenol profile of grumixama can vary significantly due to the biome where the fruit is grown [5]. The main bioactive compounds present in fruits are phenolic compounds: mainly flavonoids and ellagitannins [8,9] related to antibiofilm activity [4], the detection of quorum-sensing [10], and anti-inflammatory effects [6,9]. In addition, its flavor characteristics are related to the presence of volatile organic compounds.

Volatile compounds are substances released by foods that give off aromas and influence flavor [11,12,13,14,15], directly affecting consumer purchasing decisions. They arise from a complex mixture of secondary metabolites, including terpenes, esters, aldehydes, ketones, and alcohols. Terpenes, especially sesquiterpenes, have analgesic and antimicrobial properties [12,16]. However, there is not much knowledge regarding these compounds in grumixama.

Many extraction methods are used to analyze these substances, with an emphasis on solid-phase microextraction (SPME) [17,18]. SPME is a technique that allows for low-cost, fast, solvent-free extraction, has an affinity for numerous analytes, and is easily coupled to gas chromatography [19,20,21]. The development of the HS-SPME VOC extraction method involves evaluating many parameters such as fiber type, agitation, extraction time, and temperature [17,19,21].

For the analysis of separation and identification of these compounds, gas chromatography (GC) instruments coupled to mass spectrometry (MS) are used, preceded by the SPME technique, which consists of extracting volatile compounds through the fibers and transferring the material from the fibers through a chromatograph injector [8,17,22]. This extraction can vary according to the polarity of the extracting fiber and sample weight, as well as the time, and temperature of extraction, directly impacting the efficiency of the process. Knowledge of flavors and aromas is still a problem since the set of VOCs is specific for each species and fruit variety [8]. So, the determination of volatile compounds can contribute to the discovery of a new characteristic aroma of the species. The optimization of extraction processes is essential, as the SPME fiber coating material is crucial in obtaining a representative extraction of the volatile compounds profile [23].

Given the above, the objective of this work is to evaluate the efficiency of different fibers of SPME in the extraction of volatile organic compounds from grumixama pulp and to optimize a method to determine the characteristic volatile profile of this fruit.

## 2. Results and Discussion

In order to determine the ideal conditions for the extraction of VOCs by HS-SPME, the effect of sample weight, temperature, and extraction time for each of the fibers was evaluated. The SPME fibers, PA, DVB/CAR/PDMS, and PDMS/DVB, were analyzed and individually compared according to the sum of the peak areas prepared in the chromatograms of the 19 design tests. The relative areas of the isolated volatile compounds for each of the SPME fibers are shown in Table 1.

**Figure 1:**
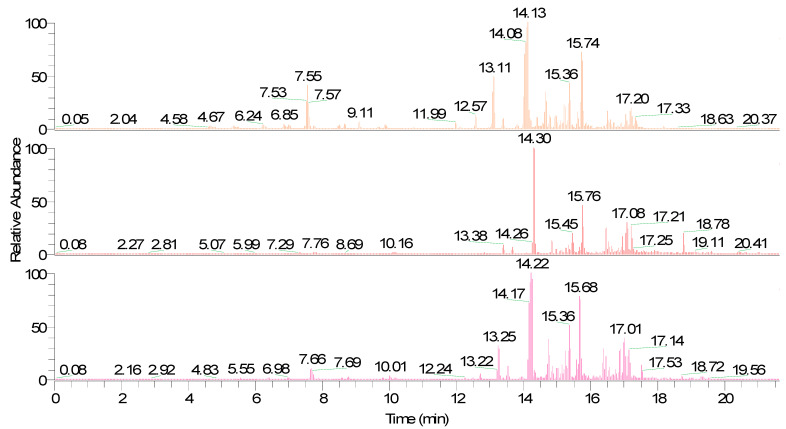
Chromatogram generated for grumixama fruits.

In the present study, it was observed that the fiber divinylbenzene/carboxen/polydimethylsiloxane (DVB/CAR/PDMS) had a better efficiency due to the larger chromatographic area observed when the grumixama pulp was subjected to the conditions of 75 °C, 2.0 g, and an adsorption time of 20 min. On the other hand, studies on the optimization of extraction conditions in fruits belonging to the Myrtaceae family, such as acerola (*Malpighia emarginata* D.C.) [17], cagaita (*Eugenia dysenterica*) [21], and cambuí (*Myrciaria floribunda*) [14] had a better efficiency for fiber polyacrylate (PA). For the pêra do cerrado, a better performance was observed for the PDMS/DVB fiber [15]. The observed behaviors emphasized that the extracted fiber’s efficiency could vary according to the matrix studied. Figure 1 shows chromatograms obtained from the studied grumixama sample, with the same conditions used with different fibers, differentiated by their colors: orange (DVB/CAR/PDMS fiber), red (PA fiber), and pink (PDMS/DVB fiber).

For the conditions that presented a better efficiency due to the larger chromatographic area, a total of 14 volatile organic compounds (VOCs) were identified by the divinylbenzene/carboxyne/polydimethylsiloxane (DVB/CAR/PDMS), six by polyacrylate fibers (PA), and seven by polydimethylsiloxane/divinylbenzene (PDMS/DVB) through solid-phase microextraction in the headspace mode (SPME-HS). The fruits of grumixama are mainly composed of sesquiterpenes.

Evaluating each individually used fiber in the *Eugenia brasiliensis*, despite the significant difference in the number of compounds, made it possible to observe that the sum of compounds in sesquiterpene fractions (85.7%, 83.3%, and 85.7% of total VOCs ) was consistently higher than the sum of compounds in monoterpene fractions (14.3%, 16.7%, and 14.3%) for DVB/CAR/PDMS, PA, and PDMS/DVB, respectively (Table 2).

An aroma is a complex mixture of many volatile compounds, whose composition is specific to each variety and varies depending on the combination of compounds, concentrations, and the perception of each compound [24]. According to Siebert et al., 2015, studies using grumixama essential oil show its composition based on the presence of monoterpenes and sesquiterpenes [25]. Thus, these results were corroborated in the present study, which also detected the presence of these chemical classes of compounds in *E. brasiliensis*.

Monoterpenes are a class of secondary metabolites found in aromatic plants with diverse food applications [26]. Pinene stands out for being commonly present in the *Eugenia* genus [27]. This compound confers sensory characteristics, such as a characteristic pine odor, which is also observed in camphene and related to a woody and citrus odor [15].

Sesquiterpenes are also the predominant volatile compounds identified in fruits of different species and are strongly linked to flavor perception and consumer acceptance [28]. In this class, we can highlight longifolene and caryophyllene. These compounds have aromatic characteristics and are important ingredients commonly used in foods [29,30].

**Figure 2:**
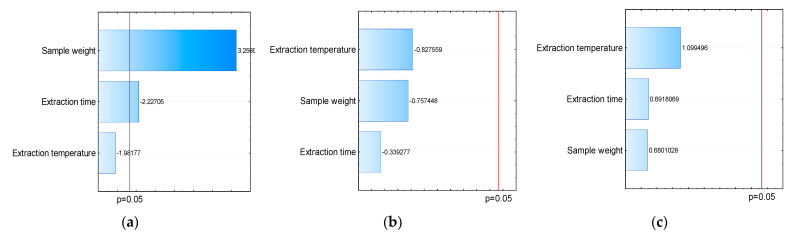
Effects of parameters: sample weight, extraction time, and extraction temperature on the extraction of volatiles using different fibers for HS-SPME-(**a**) DVB/CAR/PDMS, (**b**) PA, and (**c**) PDMS/DVB.

Several parameters influence the extraction of compounds, such as the time and temperature of extraction and the weight of the sample to be evaluated. Similarly, the amount of analytes extracted from fruit and vegetable samples depends on the nature of the fiber and the properties of the sample matrix [31]. The present study evaluates the influence of different parameters (time, temperature, and sample weight) on analyte extraction for method optimization (Figure 2).

Through Pareto’s diagram, generated from the total area, it is possible to observe that for all parameters evaluated, there was no significant difference when using PA fibers (Figure 2b) and PDMS/DVB (Figure 2c). However, a significant difference was observed when using DVB/CAR/PDMS fibers and for the variation in sample weight and extraction time (Figure 2a). The graph shows a positive correlation between the increase in weight and the extraction of analytes, so the greater the sample weight, the greater the extraction efficiency. Despite this, when we evaluated the influence of the extraction time, we observed that increasing time decreased the efficiency of analyte extraction.

**Figure 3:**
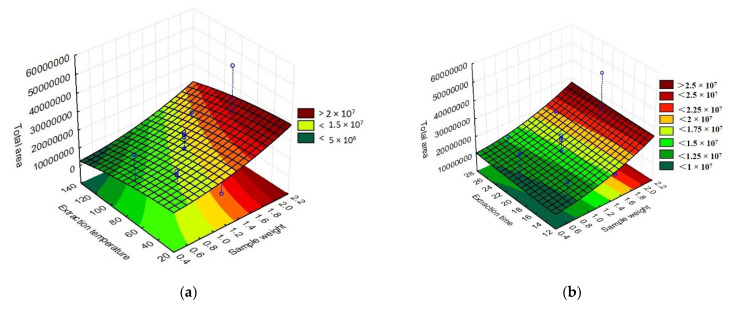

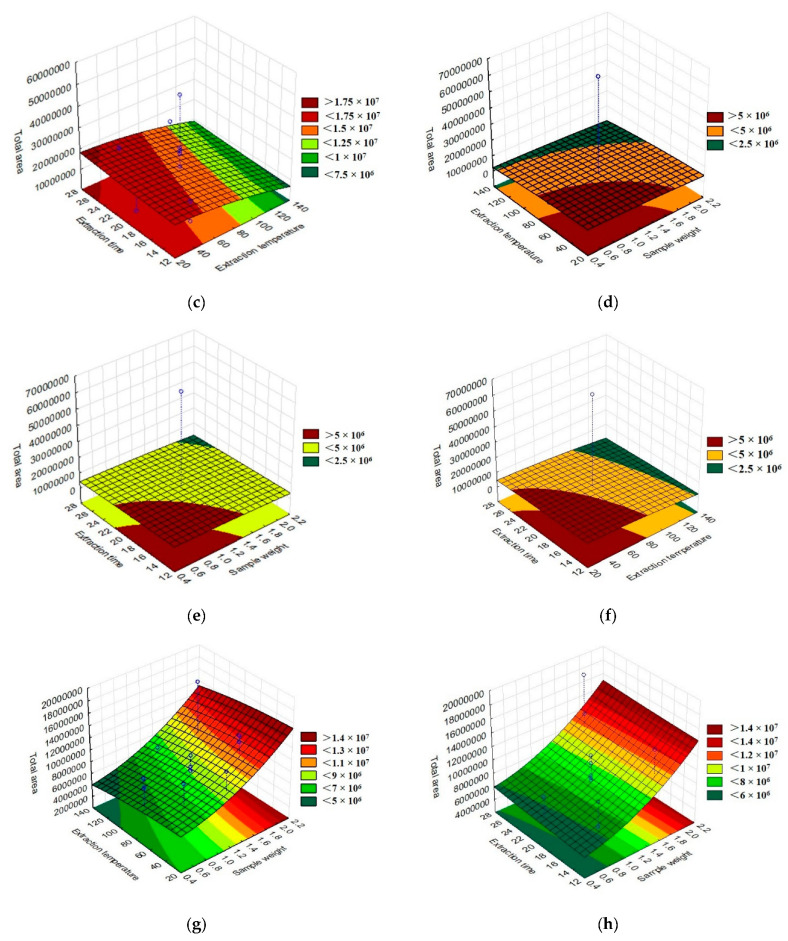

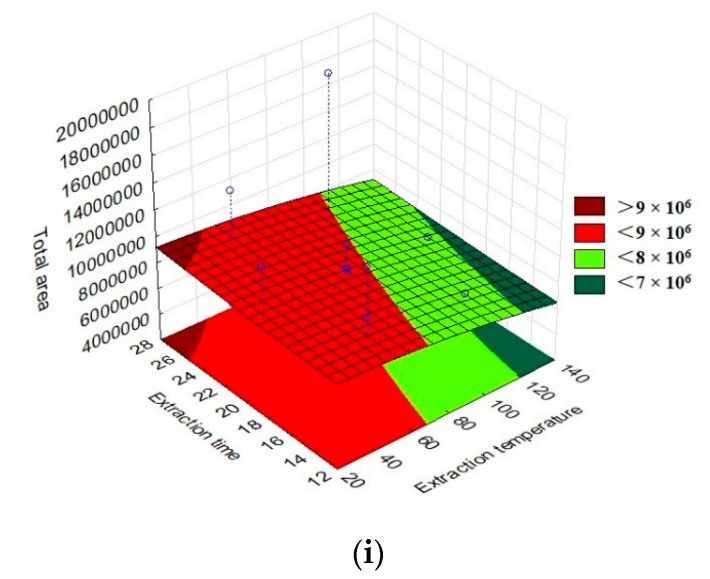
Three-dimensional response surface (RSM) graphs of the parameters time, extraction temperature, and sample weight in the extraction of volatile compounds using different fibers for HS-SPME: (**a**) DVB/CAR/PDMS extraction temperature vs. sample weight, (**b**) DVB/CAR/PDMS extraction time vs. sample weight, (**c**) DVB/CAR/PDMS extraction time vs. extraction temperature, (**d**) PA extraction temperature vs. sample weight, (**e**) PA extraction time vs. sample weight, (**f**) PA extraction time vs. extraction temperature, (**g**) DVB/PDMS extraction temperature vs. sample weight, (**h**) DVB/PDMS extraction time vs. sample weight, and (**i**) DVB/PDMS extraction time vs. extraction temperature.

The total area of the chromatographic peaks corresponding to the grumixama sample for each investigated fiber was used as a response to the presented experimental runs to obtain the response surface graphs, as shown in Figure 3. The graphs show the behavior of the extraction effectiveness of the analytes concerning the parameters evaluated.

By evaluating the response surface graphs obtained from the evaluation of the DVB/CAR/PDMS fiber data, confirmation of the influence of sample weight and time on the efficiency of analyte extraction could be observed. In addition to this, it can also be inferred that there was no significant influence on the parameters evaluated for the PA fiber. For the behavior of the graphs when using the PDMS/DVB fiber, no significant difference was observed, despite the response surface graphic showing a tendency to increase the efficiency of the process with an increase in temperature and sample weight.

## 3. Experimental Section

### 3.1. Plant Material

The grumixama pulp was obtained from the “Sítio do Bello” stores located in São Paulo, Brazil (23°27′53.94″ South and 45°42′31.88″ West), in December 2018. The pulp was stored under a temperature of −18 °C and protected from light until analysis.

### 3.2. Experimental Design

The central composite design (CCD) was used for the experimental design. It consisted of a factorial system of three factors (2^3^), with five central points, and six axial points, totaling 19 runs. The independent variables were the sample weight, adsorption temperature, and extraction time, as shown in Table 3. Statistical analyzes were performed using the Statistica v.10 software (Stat-Soft Inc., Tulsa, OK, USA) (STATSOFT, 2011) [32].

### 3.3. Extraction of Volatile Compounds

For the extraction of volatile compounds, three fibers of different polarities were used: polar polyacrylate (PA) fibers (85 μm), divinylbenzene/carboxyne/polydimethylsiloxane (DVB/CAR/PDMS) semipolar fibers (50/30 μm), and polydimethylsiloxane/divinilbenzene (PDMS/DVB) fibers (65 μm). In order to optimize the extraction conditions of the volatile organic compounds (VOCs), the samples were subjected to the experimental conditions established in the experimental design. After undergoing the experiment, the samples were refrigerated. According to the experimental method, the samples were weighed in glass vials (capacity of 20 mL) and sealed with an aluminum seal and a rubber septum. Then, they were pre-heated in a heating block, and the fibers to be used for the adsorption of the volatile substances were inserted into each flask [13,14,15,21].

### 3.4. Identification of Volatile Compounds

The identification of VOCs was performed using a gas chromatograph (Trace GC Ultra) coupled to a mass spectrometer (Polaris Q, Thermo Scientific, San Jose, CA, USA) with an ion trap analyzer using a split/splitless capillary injector.

The instrument conditions adopted were an injector temperature of 250 °C, an ion source temperature of 200 °C, and an interface temperature of 270 °C. Initially, the fiber was exposed to a temperature of 40 °C for 5 min. Then, the temperature was increased at a rate of 2.5 °C min^−1^ to 125 °C, followed by an increase of 10 °C min^−1^ at 245 °C and held for 3 min [14,21].

Data acquisition took place in full-scan mode using electronic impact ionization (EI) and a power of 70 eV, with a range from 50 to 300 *m/z*. The identification of volatile compounds was based on the mass-to-charge ratio (*m/z*) of the sample ion fragments, using each mass spectrum in the range from 50 to 300 *m/z*. Using Xcalibur software version 2.1 (Thermo Scientific, San Jose, CA, USA), a comparison was made of the mass spectra corresponding to each peak observed in the chromatogram with data obtained by the NIST library (National Institute of Standards and Technology), considering the level of similarity (reverse lookup index, RSI) greater than 600 [8,14,33].

## 4. Conclusions

Three experimental parameters were evaluated: sample weight, adsorption temperature, and extraction time (min). No significant differences were observed for PA and PDMS/DVB fibers. However, for the analysis with DVB/CAR/PDMS, there was interference regarding the sample weight and extraction time. Thus, it can be inferred that the method was efficient. Furthermore, it is noteworthy that the best experimental condition for extracting volatile organic compounds in grumixama was 75 °C, 2.0 g of pulp, and 20 min.

The profile of volatile compounds in the grumixama pulp is mainly composed of sesquiterpene compounds, representing 85.7%, 83.3%, and 85.7% for DVB/CAR/PDMS, PA, and PDMS/DVB fiber, respectively, of its composition. Fourteen volatile organic compounds (VOCs) were identified through the DVB/CAR/PDMS, six through the PA, and seven through the PDMS/DVB fiber. By analyzing the relative areas of the chromatographic peaks isolated for each SPME fiber, DVB/CAR/PDMS presented better results.

In this way, the present study presented new information about a fruit species of great importance in the Atlantic Forest, contributing to the deepening of knowledge about the sensory characteristics of grumixama pulp.

## Figures and Tables

**Table 1:** Experimental design conditions and the total sum of the chromatographic peaks obtained for each fiber by the HS-SPME-GC-MS method.

Assay	Factors			Response Variables		
	Sample Weight (g)	Adsorption Temperature (°C)	Extraction Time (min)	PA	DVB/CAR/PDMS	PDMS/DVB
1	0.80	48	14	2,800,313.178	35,673,756.71	21,632,303.63
2	1.70	48	14	3,941,457.095	44,015,733.34	25,666,945.65
3	0.80	102	14	5,408,319.333	33,243,992.42	18,340,677.84
4	1.70	102	14	6,619,351.026	29,126,870.71	23,744,873.56
5	0.80	48	26	4,708,147.715	39,677,963.22	21,550,043.39
6	1.70	48	26	6,675,326.946	63,792,454.38	28,376,523.19
7	0.80	102	26	4,239,256.12	28,867,187.53	22,588,501.49
8	1.70	102	26	7,378,696.883	38,801,629.38	35,179,099.89
9	0.5	75	20	4,299,979.278	40,662,692.46	23,106,335.27
10	2.0	75	20	6,501,985.384	70,852,087.28	24,333,026.54
11	1.25	30	20	7,252,344.778	18,319,786.5	19,673,146.52
12	1.25	120	20	1,778,008.88	16,143,761.76	20,969,695.71
13	1.25	75	10	4,276,495.736	23,722,115.99	18,184,007.64
14	1.25	75	30	8,511,420.625	35,781,050.84	27,686,545.86
15 *	1.25	75	20	7,252,344.778	46,008,220.52	20,576,090.32
16 *	1.25	75	20	6,592,025.127	44,476,047.95	35,774,028.83
17 *	1.25	75	20	4,915,434.206	35,280,027.59	26,564,485.92
18 *	1.25	75	20	7,076,964.705	31,042,244.62	24,582,273.14
19 *	1.25	75	20	7,519,311.453	37,833,823.32	23,908,051.82

SPME fibers: polyacrylate (PA), polidimetilsiloxano/divinilbenzeno (PDMS/DVB), and divinylbenzene/carboxen/polydimethylsiloxane (DVB/CAR/PDMS). * Central points.

**Table 2:** Volatile profile of fruits of *Eugenia brasiliensis*, isolated by different fibers and SPME-HS/GC–MS.

*n*°	Compound	Formula	CAS	MS/MS	Fiber		
					DVB/CAR/PDMS	PA	PDMS/DVB
**Monoterpenes**							
1	Pinene	C_10_H_16_	7785-70-8	136, 121, 93	x	-	-
2	Camphene	C_10_H_16_	79-92-5	136, 121, 95	x	x	x
**Sesquiterpenes**							
3	1H-Cyclopenta [1,3]cyclopropa [1,2]benzene, octahydro-7-methyl-3-methylene-4-(1-metylethyl)-, [3aS-(3aà,3bá,4á,7à.7aS)]-	C_15_H_24_	13744-15-5	204, 161, 120	x	-	-
4	Cubebene	C_15_H_24_	5951-67-7	204, 161, 119	x	-	-
5	Gurjunene	C_15_H_24_	489-40-7	204, 161, 119	x	-	x
6	Longifolene	C_15_H_24_	475-20-7	204, 161, 133	x	x	-
7	Cyclosativene	C_15_H_24_	22469-52-9	204, 161, 133	x	-	-
8	Guaiene	C_15_H_24_	3691-12-1	227, 161, 105	x	x	-
9	2H-2,4a-methanonaphthalene, 1,3,4,5,6,7- hexahydro-1,1,5,5-tetramethyl, (2S,4aR)-()	C_15_H_24_	1135-66-6	189, 133, 119	x	-	-
10	Muurolene	C_15_H_24_	24268-39-1	204, 161, 133	x	x	-
11	9-isopropyl-1-methyl-methylene-5-oxatricyclo [(5.4.0.0(3,8))] undecane	C_15_H_24_	-	205, 162, 121	x	-	-
12	(-)-caryophyllene-(11)	C_15_H_24_	87-44-5	281, 149, 147	x	x	-
13	Cubenol	C_15_H_26_O	21284-22-0	207, 161, 119	x	-	x
14	1,1,4a-Trimethyl-5,6-dimethylenedecahydronaphthalene	C_15_H_24_	-	205, 204, 161	x	-	-
15	Copaene	C_15_H_24_	3856-25-5	204, 119, 161	-	-	x
16	Viridiflorene	C_15_H_24_	21747-46-6	204, 161, 133	-	-	x
17	Cadina-1(10),4-diene	C_15_H_24_	16729-01-4	205, 204, 161	-	-	x
18	(4-isopropyl-1-methyl 6,7dimethylenebicyclo [3.2.1]octo-8 yl) methanol	C_15_H_24_O	-	191, 149, 135	-	-	x
19	Patchoulene	C_15_H_24_	560-32-7	204, 189, 161	-	x	-

**Table 3:** Variables used in factorial planning 2^3^ with a central component for optimizing HS-SPME conditions.

Variables	Levels of Variation		
	−1	0	+1
Sample weight (g)	0.5	1.25	3.0
Adsorption temperature (°C)	30	75	120
Extraction time (min)	10	20	30

**Source:** Authors (2022).

## Data Availability

All data are contained within the article.

## Sample Availability

Samples of grumixama are available from the authors.
